# The Avon Longitudinal Study of Parents and Children - A resource for COVID-19 research: Antibody testing results, April – June 2021

**DOI:** 10.12688/wellcomeopenres.17294.2

**Published:** 2022-01-24

**Authors:** Daniel Major-Smith, Sarah Matthews, Thomas Breeze, Michael Crawford, Hannah Woodward, Nicholas Wells, Ruth Mitchell, Lynn Molloy, Kate Northstone, Nicholas John Timpson

**Affiliations:** 1 Population Health Sciences, Bristol Medical School, University of Bristol, Bristol, UK; 2 MRC Integrative Epidemiology Unit, University of Bristol, Bristol, UK

**Keywords:** ALSPAC, Children of the 90s, birth cohort study, COVID-19, coronavirus, antibody testing, vaccination

## Abstract

The Avon Longitudinal Study of Parents and Children (ALSPAC) is a prospective population-based cohort which recruited pregnant women in 1990-1992 and has followed these women, their partners (Generation 0; G0) and their offspring (Generation 1; G1) ever since. The study reacted rapidly and repeatedly to the coronavirus disease 2019 (COVID-19) pandemic, deploying multiple online questionnaires and a previous home-based antibody test in October 2020. A second antibody test, in collaboration with ten other longitudinal population studies, was completed by 4,622 ALSPAC participants between April and June 2021.

Of 4,241 participants with a valid spike protein antibody test result (8.2% were void), indicating antibody response to either COVID-19 vaccination or natural infection, 3,172 were positive (74.8%). Generational differences were substantial, with 2,463/2,555 G0 participants classified positive (96.4%) compared to 709/1,686 G1 participants (42.1%). Of 4,199 participants with a valid nucleocapsid antibody test result (9.2% were void), suggesting potential and recent natural infection, 493 were positive (11.7%); 248/2,526 G0 participants (9.8%) and 245/1,673 G1 participants (14.6%) tested positive, respectively. We also compare results for this round of testing to that undertaken in October 2020. Future work will combine these test results with additional sources of data to identify participants’ COVID-19 infection and vaccination status.

These ALSPAC COVID-19 serology data are being complemented with linkage to health records and Public Health England pillar testing results as they become available, in addition to four previous questionnaire waves and a prior antibody test. Data have been released as an update to the previous COVID-19 datasets. These comprise: 1) a standard dataset containing *all* participant responses to all four previous questionnaires with key sociodemographic factors; and 2) individual participant-specific release files enabling bespoke research across all areas supported by the study. This data note describes the second ALSPAC antibody test and the data obtained from it.

## Introduction

At the time of writing (October 2021), the coronavirus disease 2019 (COVID-19) pandemic is over a year into its natural history. The global impact has been considerable, with over 230 million confirmed cases – and over 4.5 million deaths – to date. Despite the roll-out of vaccines, infection is still prevalent and many countries remain under some form of restrictions. More detailed information on the COVID-19 timeline in the UK can be viewed here.

Given that many people infected with severe acute respiratory syndrome coronavirus 2 (SARS-CoV-2) remain asymptomatic, antibody testing is a useful tool for assessing the prevalence of SARS-CoV-2 infection in the general population. Antibody testing is now widespread and is part of the UK government’s response to monitor the prevalence of COVID-19. Antibody testing in longitudinal population-based studies can be beneficial to objectively identify cases, validate other methods of reporting (e.g., self-reported COVID-19 status, or symptoms) and identify risk factors for SARS-CoV-2 infection using extensive pre-pandemic data and planned follow-up work. Through using multiple longitudinal studies, the UK Longitudinal Health & Wellbeing National Core Studies (LH&W NCS) were established by the UK’s Chief Scientific Advisor to conduct a program of research about COVID-19. The joint analysis of these studies will increase statistical power, particularly of low prevalence symptoms/outcomes such as post-COVID-19 syndrome (or “long COVID”), and will also increase the heterogeneity of the sample in order to allow adequately powered sub-group analysis (for example, considering outcomes of different ethnic groups or those with different socioeconomic position or in different occupation groups; see, e.g., 1– 3). Here we report results from the Avon Longitudinal Study of Parents and Children (ALSPAC) serology testing only.

ALSPAC is a unique three-generation study, comprising ‘G0’: the cohort of original pregnant women, the fathers/partners of these women; ‘G1’: the cohort of index children; and ‘G2’: the cohort of offspring of the index children. The study has a wealth of biological, genetic and phenotypic data across these generations ^4– 7^. ALSPAC has been well placed to capture information across key parts of the population during the COVID-19 pandemic – in particular the contrast between those in higher risk (the G0 cohort; mean age ~60 years) and lower risk (the G1 cohort; mean age ~29 years) groups. We have been able to collect repeat data quickly using our existing infrastructure for online data collection. So far, ALSPAC has conducted four COVID-19 questionnaires using G0 and G1 data: the first between 9 ^th^ April and 15 ^th^ May 2020 ^8^, the second between 26 ^th^ May and 5 ^th^ July 2020 ^9^, the third – with a first home-based antibody test – between 3 ^rd^ and 20 ^th^ October 2020 ^10^, and the fourth between 17 ^th^ November 2020 and 19 ^th^ March 2021 ^11^. As part of the second questionnaire, parents of G2 children also completed a questionnaire for each of their children ^12^.

The wider COVID-19 data collection in ALSPAC includes data from three main sources: self-reported data from questionnaires, data from clinical services based on linkage to medical and other records (such as Public Health England [PHE] Pillar I and II testing ^13^) and information from biological samples. The data from these sources are intended to be complementary and help address different potential research questions around COVID-19.

This data note describes the data collected via our second antibody test between 14 ^th^ April and 28 ^th^ June 2021, provides a summary of the participants who responded and details some considerations when using these data. The primary aim of the first home-based antibody test in October 2020 ^10^ was to identity how many people in the study may have been infected with SARS-CoV-2 given the asymptomatic nature of the disease in many cases. The aim of this second antibody test was again to measure infection prevalence within the study (at a later time-point; April 2021; allowing the study of the tenure of immune response), but also to identify antibody response to COVID-19 vaccination and patterns of maintained immune response in the unvaccinated. This test was administered by ten other longitudinal population studies; combined results and detailed descriptions of infected and vaccinated cases will be presented elsewhere.

## Methods

### Setting

ALSPAC is an intergeneration longitudinal cohort that recruited pregnant women residing in Avon, UK with expected dates of delivery 1 ^st^ April 1991 to 31 ^st^ December 1992 ^4, 5^. The initial cohort consisted of 14,541 pregnancies resulting in 14,062 live births and 13,988 children who were alive at 1 year of age. From the age of seven onwards, the initial sample was bolstered with eligible cases who had originally failed to join the study and there were subsequently 14,901 children alive at 1 year of age following this further recruitment ^6^. Please note, the study website contains details of all the data that is available through a fully searchable data dictionary and variable search tool.

In response to the COVID-19 pandemic it was necessary to develop a data collection strategy which was practical, would yield data quickly and could be updated and repeated if necessary. For these reasons, we initially chose to use an online-only data collection approach for this, restricting our invites to those participants with a valid email address (and coordinated with a systematic communications/outreach campaign to obtain updated information from participants). This approach was again followed for the second antibody test, with the consent and screening form emailed to participants for online completion. The online consent questionnaire was developed and deployed using REDCap (Research Electronic Data CAPture tools ^14^); a secure web application for building and managing online data collection exercises, hosted at the University of Bristol.

### Invitation and reminder strategy for second antibody test

**Figure 1.**
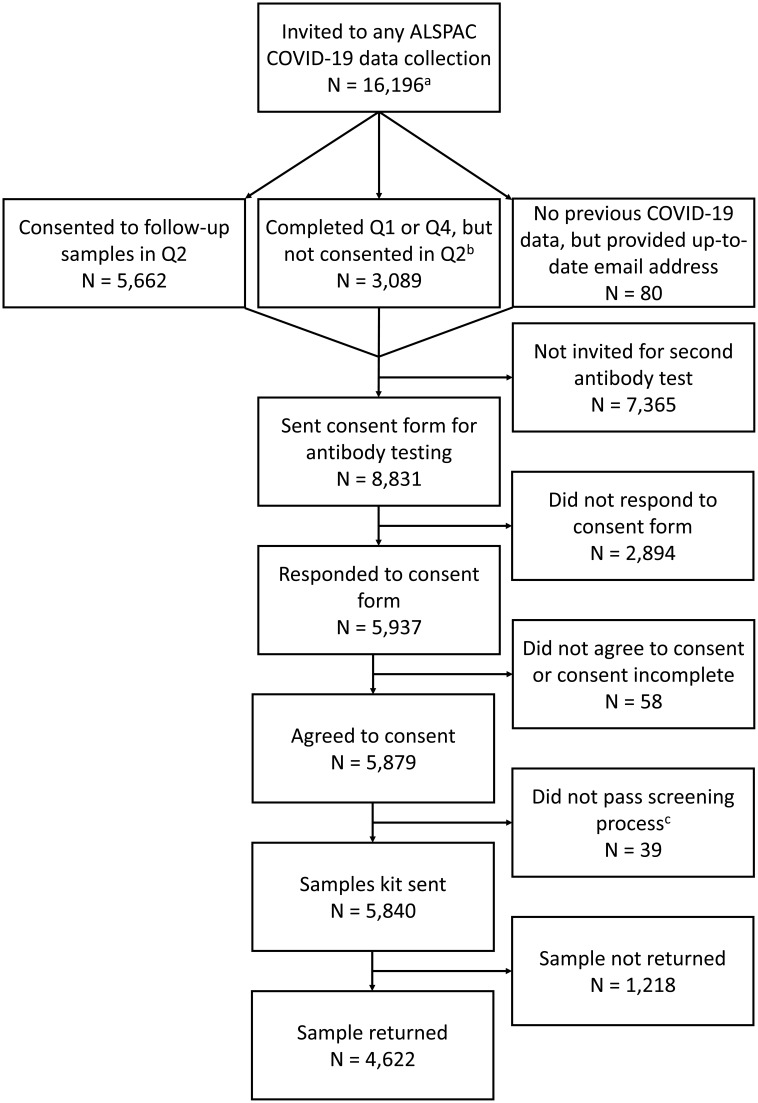
Flow chart showing the number of participants invited and reasons for not returning a sample. Q = questionnaire. ^a^ This is the total number of participants invited to any of the ALSPAC COVID-19 data collections (i.e., questionnaires 1 through 4, plus the second antibody test), and includes all participants who were still alive, had not withdrawn consent to be contacted and had a valid email address (although some G1 offspring with valid home addresses, but not valid email addresses, were invited to the COVID4 questionnaire; see the COVID Q4 data note for more details ^11^). Of this 16,196 total, 4,883 were G0 mothers, 1,894 were G0 fathers/partners, 9,132 were G1 offspring, and 287 were G1 offspring partners. ^b^ Note that 197 G1 offspring who completed the COVID4 questionnaire but who did not have a valid email address – and hence were not invited to complete a second antibody test – are not included in this total. ^c^ These participants were excluded because they did not answer or did not pass the bleeding disorder screening, they lived outside the UK, or did not confirm their postcode.

Participants who met any of the following criteria were invited to complete the second antibody test ( Figure 1): 1) Participants who answered that they were happy to be contacted about future research projects involving testing or taking of biological samples as part of the second COVID-19 ALSPAC questionnaire; 2) Participants who completed either the first or fourth COVID-19 questionnaire, but not the second; or 3) Participants who were not invited to complete any of the previous ALSPAC COVID-19 questionnaires, but had recently provided an up-to-date email address to the ALSPAC administration team. Participants were not contacted if our administrative database records indicated that they were deceased, had withdrawn from the study, had declined further contact or had declined to complete questionnaires.

Between 22 ^nd^ March and 1 ^st^ April 2021, an initial email was sent to participants asking them to read a participant information sheet and instruction booklet (which included a link to a brief video), which contained details on the aims of the research, what was involved, and the risks of participating. Participants were asked to complete an online REDCap consent form to confirm that they had read and understood the information provided, had the opportunity to ask any questions about the research, and that they agreed to participate in the study. Participants were asked a screening question about their coagulation disorders; any participant who reported an increased risk of bleeding was told that they would not be able to take part. Participants were also asked to verify their date of birth and current postcode, the latter to ensure the blood sample collection kit was sent to the correct address and meant we could exclude participants living outside of the UK. This online consent form was live until 1st April 2021, and participants were unable to complete a consent form after this date.

A total of eleven studies took part in this data collection exercise, coordinated by University College London ( Table 1). Thriva, the company providing the antibody tests, administer many of the NHS ‘at home’ antibody tests and are approved by the Department for Health and Social Care.

**Table 1.** Summary of the 11 longitudinal population studies involved in the wider serology data collection.

Longitudinal Populations Study	Geography	Response rate (%)	Age (mean years)	Unique characteristics
*Millennium Cohort Study*	Nationwide	Child: 1135/1767 (64%) Parent: 2255/3229 (70%)	20	Birth cohort
*ALSPAC (Avon Longitudinal Study of Parents and* * Children)*	Bristol	4622/5840 (79%)	G0 – 28 G1 – 58	Birth cohort with repeat serology
*Next Steps*	Nationwide	1260/2098 (60%)	31	Adolescence cohort
*Understanding Society*	Nationwide	6667/10157 (65%)	49	Household
*British Cohort Study 1970*	Nationwide	2538/3765 (67%)	51	Birth cohort
*TwinsUK*	Nationwide	4258/4915 (87%)	53	Repeat serology
*EXCEED (Extended Cohort for E-health,* * Environment and DNA)*	Leicester	2364/2853 (83%)	62	Repeat serology
*1958 NCDS (National Child Development Study)*	Nationwide	3211/4160 (77%)	63	Birth cohort
*ELSA (English longitudinal Study of Ageing)*	Nationwide	3472/6955 (50%)	70	Older population
*SABRE (Southall and Brent Revisited)*	London	174/259 (67%)	74	Ethnic differences
*1946 NSHD (National Survey of Health and* * Development)*	Nationwide	898/1125 (80%)	75	Birth cohort

Eligible participants were sent a blood sample collection kit through the post on 8 ^th^ April 2021. A letter accompanying the kit included detailed instructions on how to collect the blood sample (0.5ml) and how to send the resulting sample to Thriva, who analysed the samples (see the ‘antibody test’ section below). Reminders were sent on 13 ^th^ April 2021. All results were returned to ALSPAC from Thriva by 28 ^th^ June 2021. Participants were informed of their result by ALSPAC. Participants were told they had a ‘positive’ result if their test was positive to either nucleocapsid or spike antibodies (see the ‘antibody test’ section below for more information on these tests). If the result was ‘void’, participants were informed. We did not provide any incentive for participating in this study. Participants incurred no cost in providing a blood sample, with the blood sample collection kit including all necessary instructions and equipment, and a cardboard box with a pre-paid form to return the samples in. For full details of the protocol, including instructions sent to participants regarding the blood collection procedure, see the extended data ^15^.

### Antibody test

The pseudonymised blood sample was collected in dedicated tubes and sent to Thriva where it was processed (centrifuged to separate the serum) for COVID-19 antibody testing. The testing utilised the CE marked capillary blood Roche “Elecsys® Anti-SARS-CoV-2” ^16^ and “Elecsys® Anti-SARS-CoV-2 S” ^17^ assays.

The Elecsys® Anti-SARS-CoV-2 is an immunoassay for the qualitative detection of antibodies (including IgG) to the SARS-CoV-2 nucleocapsid antigen. The Elecsys® Anti-SARS-CoV-2 S is an immunoassay for the quantitative determination of antibodies to the spike protein of SARS-CoV-2. Specifically, the test targets antibodies which are directed against the particular region of the viral spike protein responsible for binding to the host cell receptor, which is required for the virus to enter the host cell. Both tests are intended as an aid in the determination of the immune reaction to SARS-CoV-2 and the presence and level of such antibodies could signal whether a person has been already infected and potentially developed immunity to the virus. The COVID-19 vaccines that have been administered in the UK generate immune responses against the spike antigen of SARS-CoV-2. Therefore, the two assays used in this study can distinguish between an immune response to a natural infection with Elecsys® Anti-SARS-CoV-2 and, although not exclusively, an immune response to vaccine with Elecsys® Anti-SARS-CoV-2 S. We refer to these from now on as the N (nucleocapsid) response and the S (spike) response, respectively.

Thriva processed the samples and securely returned results to ALSPAC periodically. Under the Health Protection Regulations 2020, there is a legal requirement for non-identifiable COVID-19 results and associated demographic data to be reported to Public Health England (PHE). These data were therefore provided to PHE by ALSPAC in an anonymised form at the end of data collection.

The results returned from Thriva consist of two assays as described above: the nucleocapsid infection response (in ‘cut-off index’ [COI] units), which suggests antibody response to natural SARS-CoV-2 infection, and secondly a spike protein response (in U/ml units), which can be induced either by vaccination or by natural infection. For the N assay, values less than 1 COI have been coded as ‘negative’, and values greater or equal to 1 COI have been coded as ‘positive’. For the S assay, values less than 0.80 U/ml have been coded as ‘negative’, and values greater or equal to 0.80 as ‘positive’.

It is important to note that interpretation of a single antibody test result (either N or S) may be ambiguous as the S test can indicate either natural infection or vaccine spike response, while the results of the N test cannot be attributed to previous COVID-19 infection with certainty as nucleocapsid antibody response may wane over time ^18– 20^. To identify infection and vaccination status, both N and S test results should be therefore interpreted in combination, ideally with additional information such as previous antibody test results and vaccination status and dates ( Centers for Disease Control and Prevention [CDC] antibody test guidelines; Twins UK ‘Interpreting serological response to CARS-CoV-2’ working document, Personal Communication). At present, ALSPAC does not possess data on participants’ vaccination status and dates, although this is currently being collected; as such, for the purposes of this data note and to describe these data, we largely treat the N and S antibody response tests separately, while noting that future work aiming to explore and analyse natural COVID-19 infection and vaccination cases in greater detail should interpret these results in tandem, along with additional forthcoming data on vaccinations and other sources of information. Future work from the LH&W NCS, combining data from all 11 longitudinal population studies, will describe these infection and vaccination cases in greater detail.

## Key results

### Response rate

A total of 8,831 consent form invitations were sent out ( Figure 1). Of these, 5,840 participants (66%) completed the consent form, agreed to participate, passed the blooding disorder and address screening (39 participants did not pass this screening process) and were sent a kit. Of those sent a kit, 4,622 returned a sample (79% of those sent a kit; 52% of invited sample). Samples were received by Thriva between 14 ^th^ April and 25 ^th^ June 2021. 4,466 samples (96.6% of those returned) were received by Thriva in April, with 146 (3.2%) and 10 (0.2%) in May and June, respectively.

Invitation and response rate by cohort structure is provided in Table 2. Response rates for both being sent a kit and returning a sample were higher among the older G0 generation (3,187/4,374 [73%] sent a kit, of which 2,772 [87%] returned a sample) relative to the younger G1 generation (2,653/4,457 [60%] sent a kit, of which 1,850 [70%] returned a sample). Female participants were more likely to complete and agree to the consent form and be sent a kit (4,223/6,142 [69%] females vs 1,617/2,689 [60%] males), although return rates of those who were sent a kit were similar between the sexes (80% females vs 78% males).

**Table 2.** Number of participants who were eligible, who consented and were sent a samples kit, and who returned a sample.

Cohort Group	Eligible ^1^	Sent a samples kit ^2^	Returned a sample ^3^
G0 Mothers	3,181	2,357 (74%)	2,055 (65% invited; 87% sent a kit)
G0 Fathers/partners	1,193	830 (70%)	717 (60% invited; 86% sent a kit)
G1 Offspring daughters	2,900	1,823 (63%)	1,270 (44% invited; 70% sent a kit)
G1 Offspring sons	1,435	749 (52%)	521 (36% invited; 70% sent a kit)
G1 Offspring partners (female)	61	43 (70%)	33 (54% invited; 77% sent a kit)
G1 Offspring partners (male)	61	38 (62%)	26 (43% invited; 68% sent a kit)
**TOTAL**	**8,831**	**5,840 (66%)**	**4,622 (52% invited; 79% sent a kit)**

^1 ^Eligibility criteria: 1) Participants who were happy to be contacted about future research projects involving biological samples as part of the second coronavirus disease 2019 (COVID-19) ALSPAC questionnaire; 2) Participants who completed either the first or fourth COVID-19 questionnaire, but not the second; or 3) Participants who were not invited to complete any of the previous ALSPAC COVID-19 questionnaires, but recently provided an up-to-date email address to the ALSPAC administration team. ^2^ Proportions of those invited (i.e., eligible). ^3^ Proportions of those invited (i.e., eligible) and sent a samples kit

### Participant demographics

Characteristics of responders according to key variables that have been released with the complete dataset can be seen in Table 3. The sample who responded were predominantly white (> 95%) and the majority had at least A-level qualifications (optional exams in the UK sat at the age of 18 years), with 55% of G0 mothers, 71% of G0 partners/fathers, 81% of G1 offspring and 71% of G1 partners in this category. G0 partners/fathers were three years older on average than G0 mothers (61.6 years vs 58.9 years), and G1 partners were on average two years older than G1 offspring (30.7 years vs 28.6 years).

**Table 3.** Summary of key characteristics for those who returned a sample for the second antibody test; n (%) for categorical variables or mean (standard deviation [sd]) for continuous variables. As some variables contain missing data, the sample size for each characteristic is given in brackets after the % (for categorical variables) or sd (for continuous variables). Total sample sizes for each cohort are given on the top row. BMI=body mass index; BP=blood pressure.

Key Characteristic	G0 Mothers (total *n* = 2,055)	G0 Fathers/ partners (total *n* = 717)	G1 Offspring (total *n* = 1,791)	G1 Offspring partners (total *n* = 59)
Age (years)	58.9 (4.32; *n* = 2,055)	61.6 (4.71; *n* = 717)	28.6 (0.54; *n* = 1,791)	30.7 (3.94; *n* = 59)
Latest BMI ^1^	26.2 (4.80; *n* = 1,750)	27.4 (3.95; *n* = 582)	24.6 (5.03; *n* = 1,577)	27.3 (4.82; *n* = 38)
Latest Systolic BP ^1^	119.3 (13.95; *n* = 1,747)	132.9 (13.04; *n* = 588)	115.6 (10.96; *n* = 1.564)	116.2 (12.49; *n* = 31)
Latest Diastolic BP ^1^	70.6 (9.19; *n* = 1,747)	77.5 (8.66; *n* = 588)	66.8 (7.56; *n* = 1,564)	65.9 (10.74; *n* = 31)
Education level ^2^ ≥A level	1,075 (54.7%; *n* = 1,964)	481 (71.2%; *n* = 676)	1,109 (80.8%; *n* = 1,373)	15 (71.4%; *n* = 21)
Ethnicity ^3^ White	2,018 (98.5%; *n* = 2,049)	705 (98.6%; *n* = 715)	1,698 (95.6%; *n* = 1,776)	Not available

^1^Data taken from the most recent clinic that individual attended where available ^2^Data taken from pregnancy questionnaires for G0 and from most recent questionnaire for G1 where available ^3^Data taken from pregnancy questionnaires or COVID4 questionnaire (if missing from pregnancy questionnaire) for all

### Antibody test results

Table 4 presents a summary of the test results. Of the 4,622 participants who returned a sample, 9.1% and 8.2% of results were ‘void’ in the N and S response assays, respectively. 373 samples (8.1%) were void on both assays, 50 (1.1%) were void only for N, 8 (0.2%) were void only for S response, and 4,191 (90.7%) had valid tests for both measures. The main reasons for void results included insufficient blood, haemolysed samples and delays of more than seven days between the date the sample was taken and the date it arrived in the lab (either due to postal delays, or delays in participants posting the sample after taking it).

**Table 4.** Summary of coronavirus disease 2019 (COVID-19) N (nucleocapsid), S (spike) response and combined N and S test results.

Test result	G0 Mothers ( *n* = 2,055)	G0 Fathers/ partners ( *n* = 717)	G1 Offspring ( *n* = 1,791)	G1 Offspring partners ( *n* = 59)	Total ( *n* = 4,622)
***COVID-19 N (nucleocapsid) antibody response***					
Positive (COI ≥ 1) ^1^	185 (9.8%)	63 (10.0%)	239 (14.8%)	6 (10.9%)	493 (11.7%)
Negative (COI < 1) ^1^	1,709 (90.2%)	569 (90.0%)	1,379 (85.2%)	49 (89.1%)	3,706 (88.3%)
Void ^2^	161 (7.8%)	85 (11.9%)	173 (9.7%)	4 (6.8%)	423 (9.2%)
***COVID-19 S (spike) antibody response***					
Positive (U/ml ≥ 0.80) ^1^	1,847 (96.5%)	616 (96.1%)	684 (41.9%)	25 (45.5%)	3,172 (74.8%)
Negative (U/ml < 0.80) ^1^	67 (3.5%)	25 (3.9%)	947 (58.1%)	30 (54.6%)	1,069 (25.2%)
Void ^2^	141 (6.9%)	76 (10.6%)	160 (8.9%)	4 (6.8%)	381 (8.2%)
**Combined COVID-19 N and S antibody responses ^3^**					
N + S negative ^1^	66 (3.5%)	25 (4.0%)	940 (58.3%)	30 (54.5%)	1,061 (25.3%)
S positive + N negative ^1^	1,640 (86.8%)	543 (86.1%)	436 (27.0%)	19 (34.5%)	2,638 (63.0%)
N + S positive ^1^	184 (9.7%)	63 (10.0%)	236 (14.6%)	6 (10.9%)	489 (11.7%)
Void ^2, 4^	165 (8.0%)	86 (12.0%)	176 (9.8%)	4 (6.8%)	431 (9.3%)

^1^ Percentages based on those with a valid test result (i.e., not ‘void’). ^2^ Percentages based on total number who returned a sample. ^3^ Note that three G1 offspring cases where N is positive and S is negative have been removed for the combined results. The total number of participants for this analysis is therefore 4,619 (rather than 4,622), while the total number of G1 offspring is 1,788 (rather than 1,791). ^4^ For the combined results, if either N or S result was void, the combined result was coded as void.

**Figure 2.**
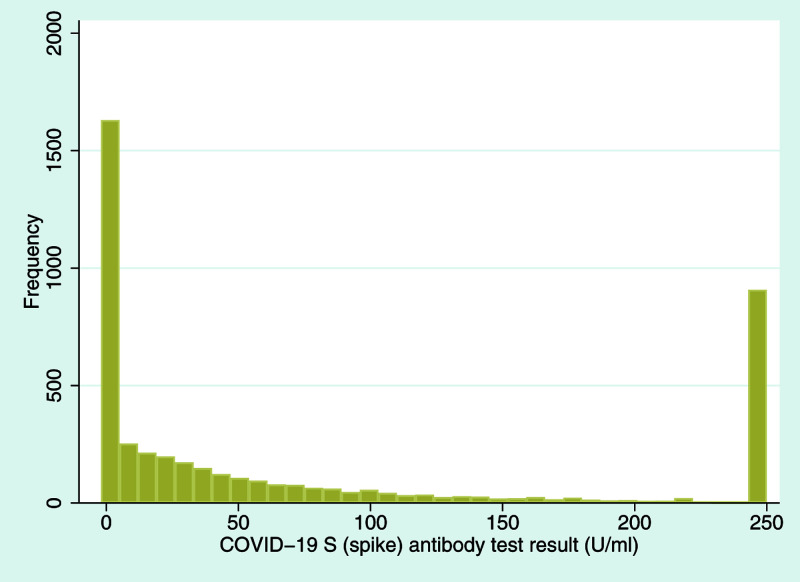
Histogram of coronavirus disease 2019 (COVID-19) S (spike) antibody test results. COVID-19 S (spike) antibody response, indicating either natural infection or vaccine response (U/ml; mean = 83.55, standard deviation = 97.79, median = 34.41, interquartile range = 0.60 to 159.70, minimum value = 0.40, maximum value = 250; note that the upper limit for this test is 250 U/ml, hence the spike at this value).

Taking the spike antibody response first, 74.8% of participants had a positive result (defined as a U/ml value ≥ 0.80). This varied by generation, with G0 participants much more likely to have a positive S antibody test result (G1: 709/1,686 positive [42.1%]; G0: 2,463/2,555 positive [96.4%]; *χ* ^2^(1, *n* = 4,241) = 1591, *p*<0.001). Given that the vaccination program in the UK at the time had focused on older and vulnerable members of the population, and that current vaccines target the SARS-CoV-2 spike protein, this difference is perhaps not surprising. A histogram of the S antibody test results is displayed in Figure 2 (note that values of the S response test were capped at 250 U/ml as a serial dilution to see the lower ranges, hence the bimodal appearance of the histogram). Compared to nationwide antibody statistics compiled by the UK’s Office for National Statistics (ONS) on approximately the same dates (mid/late April 2021) in England, the proportion of positive spike protein antibodies reported here (~75%) is similar, albeit slightly higher, than that reported by the UK Health Security Agency sero-surveillance programme using the same Roche S antibody test (~65–70%). These ALSPAC results are also higher than the Coronavirus Infection Survey statistics, which used an ELISA test for immunoglobulins IgG based on SARS-CoV-2 trimeric spike protein, where antibody rates were ~55–60%.

Levels of nucleocapsid antibody response were considerably lower, with 11.7% of participants having a positive test result (defined as a COI value ≥ 1). This again varied by generation, with G1 participants more likely to have a positive N antibody test result (G1: 245/1,673 positive [14.6%]; G0: 248/2,526 positive [9.8%]; *χ* ^2^(1, *n* = 4,199) = 22.62, *p*<0.001). A histogram for the N antibody test results has not been displayed as most values are ~0, with counts ≥ 1 difficult to read.

The combined results of the N and S antibody tests are also presented in Table 4, with results grouped into three categories: N and S negative (likely indicating no natural infection and no vaccination); N negative and S positive (likely indicating either no natural infection but had vaccination, or had natural infection but N antibody response waned [with or without vaccination]) and N and S positive (likely indicating natural infection, but uncertain about vaccine status). Note that three individuals were N positive and S negative, but have been excluded here. Of those with valid data, 25.3% were negative to both N and S antibodies, 63.0% were negative to N antibody but positive to S antibodies, and 11.7% were positive to both N and S antibodies. As with the separate N and S results above, this varied greatly by generation ( *χ* ^2^(2, *n* = 4,188) = 1759, *p*<0.001): of 2,521 G0 participants with data, 91 (3.6%) were N and S negative, 2,183 (86.6%) were N negative and S positive, and 247 (9.8%) were N and S positive; in contrast, of 1,667 G1 participants with data, 970 (58.2%) were N and S negative, 455 (27.3%) were N negative and S positive, and 242 (14.5%) were N and S positive.

A breakdown of differences between individuals with positive and negative test results, within each generation, for each of the key sociodemographic characteristics in Table 3 is reported in Table 5 (for the N assay) and Table 6 (for the S assay). For the N response, younger participants, those without A-level or higher qualifications, and participants with a higher BMI were associated with having a positive N antibody response in the G0 generation. No associations were evident in the G1 generation. For the S response, older participants, those with A-level or higher qualifications, participants with a higher BMI, and participants with a white ethnicity background were more likely to have a positive S response in the G0 generation. In the G1 generation, female participants and those with higher BMI were more likely to have a positive S response.

**Table 5.** Coronavirus disease 2019 (COVID-19) N (nucleocapsid) antibody test result according to key variables, stratified by generation. Differences between positive and negative test results were assessed using chi-squared test for categorical variables and t-test for continuous variables; n (%) for categorical variables or mean (standard deviation [sd]) for continuous variables. A positive result is defined as an N response value of COI ≥ 1. Participants with ‘void’ test results have been excluded here. BMI=body mass index; BP=blood pressure.

	Positive N antibody result	Negative N antibody result	*p*-value
**Age (years)** G0 ( *n* = 2,526) G1 ( *n* = 1,673)	58.4 (4.4; *n* = 248) 28.7 (0.8; *n* = 245)	59.7 (4.6; *n* = 2,278) 28.7 (1.0; *n* = 1,428)	<0.001 0.755
**Sex** G0 ( *n* = 2,526) Male Female G1 ( *n* = 1,673) Male Female	63 (10.0%) 185 (9.8%) 71 (14.4%) 174 (14.7%)	569 (90.0%) 1,709 (90.2%) 421 (85.6%) 1,007 (85.3%)	0.883 0.873
**Education ^1^** G0 ( *n* = 2,405) Lower than A levels A levels or higher G1 ( *n* = 1,270) Lower than A levels A levels or higher	120 (12.2%) 115 (8.1%) 36 (14.9%) 142 (13.8%)	860 (87.8%) 1,310 (91.9%) 205 (85.1%) 887 (86.2%)	0.001 0.647
**BMI ^2^** G0 ( *n* = 2,145) G1 ( *n* = 1,463)	27.9 (5.2; *n* = 203) 24.3 (4.4; *n* = 222)	26.3 (4.4; *n* = 1,942) 24.7 (5.1; *n* = 1,241)	<0.001 0.193
**Systolic BP ^2^** G0 ( *n* = 2,143) G1 ( *n* = 1,446)	124.4 (14.0; *n* = 202) 115.8 (12.2; *n* = 221)	122.4 (15.1; *n* = 1,941) 115.6 (10.9; *n* = 1,225)	0.061 0.750
**Diastolic BP ^2^** G0 ( *n* = 2,143) G1 ( *n* = 1,446)	73.1 (9.1; *n* = 202) 66.2 (7.9; *n* = 221)	72.1 (9.6; *n* = 1,941) 66.9 (7.6; *n* = 1,225)	0.129 0.248
**Ethnicity ^3^** G0 ( *n* = 2,518) White Other than white G1 ( *n* = 1,604) White Other than white	241 (9.7%) 5 (13.2%) 225 (14.7%) 13 (19.1%)	2,239 (90.3%) 33 (86.8%) 1,311 (85.4%) 55 (80.9%)	0.503 0.310

^1^Data taken from pregnancy questionnaires for G0 and from most recent questionnaire for G1 where available

^2^Data taken from the most recent clinic that individual attended where available

^3^Data taken from pregnancy questionnaires or COVID4 questionnaire (if missing from pregnancy questionnaire) for all

**Table 6.** Coronavirus disease 2019 (COVID-19) S (spike) response antibody test result according to key variables, stratified by generation.

	Positive S antibody result	Negative S antibody result	*p*-value
**Age (years)** G0 ( *n* = 2,555) G1 ( *n* = 1,686)	59.7 (4.5; *n* = 2,463) 28.8 (1.2; *n* = 709)	56.3 (4.8; *n* = 92) 28.6 (0.8; *n* = 977)	<0.001 0.048
**Sex** G0 ( *n* = 2,555) Male Female G1 ( *n* = 1,686) Male Female	616 (96.1%) 1,847 (96.5%) 175 (35.2%) 534 (44.9%)	25 (3.9%) 67 (3.5%) 322 (64.8%) 655 (55.1%)	0.638 <0.001
**Education ^1^** G0 ( *n* = 2,434) Lower than A levels A levels or higher G1 ( *n* = 1,277) Lower than A levels A levels or higher	942 (94.8%) 1,406 (97.6%) 109 (45.0%) 429 (41.5%)	52 (5.2%) 34 (2.4%) 133 (55.0%) 606 (58.5%)	<0.001 0.308
**BMI ^2^** G0 ( *n* = 2,174) G1 ( *n* = 1,476)	26.5 (4.5; *n* = 2,102) 25.2 (5.6; *n* = 631)	25.1 (4.1; *n* = 72) 24.3 (4.5; *n* = 845)	0.014 <0.001
**Systolic BP ^2^** G0 ( *n* = 2,172) G1 ( *n* = 1,459)	122.7 (15.0; *n* = 2,100) 115.2 (11.1; *n* = 624)	120.2 (13.5; *n* = 72) 115.9 (11.0; *n* = 835)	0.153 0.285
**Diastolic BP ^2^** G0 ( *n* = 2,172) G1 ( *n* = 1,459)	72.2 (9.6; *n* = 2,100) 67.2 (7.9; *n* = 624)	71.1 (9.1; *n* = 72) 66.5 (7.4; *n* = 835)	0.331 0.118
**Ethnicity ^3^** G0 ( *n* = 2,547) White Other than white G1 ( *n* = 1,604) White Other than white	NA (>96.0%) ^4^ NA (>89.0%) 646 (41.8%) 30 (43.5%)	NA (<4.0%) NA (<11.0%) 901 (58.2%) 39 (56.5%)	0.020 0.777

^1^Data taken from pregnancy questionnaires for G0 and from most recent questionnaire for G1 where available ^2^Data taken from the most recent clinic that individual attended where available ^3^Data taken from pregnancy questionnaires or COVID4 questionnaire (if missing from pregnancy questionnaire) for all ^4^N/A: Not Applicable - actual numbers withheld due to small cell counts and potential disclosure risk (cell counts < 5).

### Comparing first and second antibody test results

As an illustrative example using these serology test results in tandem with other sources of data, we examined agreement in infection status between these tests to explore how the second antibody test results compare to the first test conducted in October 2020. As our focus here is on natural infection, rather than potential vaccine response, we only look at the N assay, and not the S assay. It is important to bear in mind that a direct comparison of these results is difficult for many reasons. First, they are different tests, so may not be directly comparable (the first was a lateral flow test [LFT], while the second was an electrochemiluminescence immunoassay [ECLIA] test). LFTs tend to have high specificity (correctly ruling out infection in non-infected individuals), but lower sensitivity (correctly identifying infection in infected individuals ^21^). The LFT used for the first ALSPAC home-based antibody test ^10^ was the Una Health and Fortress Diagnostics test ^22^, which, compared to reference tests, had 84% sensitivity and 98.6% specificity ^23^. For the ECLIA tests used for the second ALSPAC serology tests, sensitivities after at least 14 days post-diagnosis after a positive PCR test are much higher, at 100% for the Elecsys® Anti-SARS-CoV-2 assay ^16^ and 98.8% for the Elecsys® Anti-SARS-CoV-2 S assay ^17^ (although prior to 14 days post-PCR confirmation sensitivities are lower due to a lag in the antibody response). Specificities are 99.81% and 99.98% for Elecsys® Anti-SARS-CoV-2 and Elecsys® Anti-SARS-CoV-2 S, respectively. There is therefore a greater risk of false negatives in the first ALSPAC serology test. Second, the tests were conducted at different times, meaning that participants could have been negative at the first test – if not a false negative – and positive for the second if they contracted the virus after October 2020. Additionally, due to waning antibody response over time, especially for N antibodies, it is also possible that participant’s infection prior to October 2021 may have been positive for the first test and negative for the second (although the extent of this waning response does appear variable, potentially due to both individual characteristics and the antibody test used ^18– 20^). The S antibody response is generally longer-lasting than the N response, but as the S response occurs to both natural infection and vaccination, without additional vaccination data it was impossible to use both N and S results together to identify potential COVID-19 cases.

Nonetheless, with these caveats in mind, in the whole ALSPAC cohort we observed that 8% of participants who were negative for the first test had a positive N antibody result for the second (with 92% negative for both), while 24% of participants were positive for the first test but had a negative N antibody for the second (with 76% positive for both); these results were broadly similar between the G0 and G1 generations, although G1s were more likely to have a positive second result following a negative first result ( Table 7).

**Table 7.** Comparing the first and second ALSPAC coronavirus disease 2019 (COVID-19) N (nucleocapsid) antibody test results. Results are presented for the whole ALSPAC cohort, as well as stratified by generation. For the first antibody test, a positive result is defined as either an ‘IgG positive’ or ‘IgG and IgM positive’ result, while a negative result is defined as either a ‘negative’ or ‘IgM positive’ result (‘invalid’, ‘can’t tell’ and ‘not sure’ results have been coded as missing). For the second antibody test, a positive result is defined as an N response value of COI ≥ 1; participants with ‘void’ test results have been excluded here.

		Second antibody N test result					
		Whole ALSPAC cohort		Generation 0		Generation 1	
		Positive	Negative	Positive	Negative	Positive	Negative
**First antibody ** **test result**	**Positive**	100 (76.3%)	31 (23.7%)	46 (76.7%)	14 (23.3%)	54 (76.1%)	17 (23.9%)
	**Negative**	238 (8.3%)	2,644 (91.7%)	122 (6.7%)	1,694 (93.3%)	116 (10.9%)	950 (89.2%)

## Strengths and limitations of the data

This data collection has a number of strengths. First, ALSPAC has been able to respond rapidly to the on-going pandemic situation, and now has four sets of questionnaire data ^8– 11^, two antibody tests ^10^, and linkage to PHE Pillar I and II test results ^13^. In addition to providing a longitudinal perspective on the developing pandemic, these multiple sources of COVID-19 infection allow us to triangulate this data to better identify ‘true’ COVID-19 cases (using self-reported COVID-19 cases, cases based on combinations of symptoms, antibody testing, and PHE testing ^13^). When combined with ALSPAC’s longitudinal COVID-19 data, these serology results can also be used to explore potential timings of COVID-19 infection (based on symptoms), the prevalence of asymptomatic infections, and agreement between the various case definitions. Additionally, as this antibody test was embedded within a longitudinal population study, these results can be analysed to explore the pre-pandemic risk factors associated with COVID-19 infection in the community.

Second, this antibody test provides an indication of the prevalence of COVID-19 infection at the time the antibody tests were taken in this population (young adults born in the early 1990s in the Bristol area and their partners [G1], and the parents of these G1 children [G0]). These results are not representative of the wider UK population, or of the Bristol-based population at other times.

Third, the current antibody tests assessed both N (nucleocapsid) and S (spike) proteins, which together – and with additional information from vaccination status and dates – can provide a fine-grained picture of COVID-19 infection and vaccination status in this population. Due to a current lack of data on vaccination status and dates (currently being collected), in this paper we have largely treated the N and S assays separately to describe the data collected and to illustrate the types of analyses that can be conducted. However, we reiterate that in future these assays should be treated in conjunction to determine case definitions and differentiate between antibody response due to natural infection and vaccination, particularly because natural infection will engage both nucleocapsid and spike protein antibodies. Additionally, both antibody assays have been correlated to pseudo-neutralisation assay with an overall 87.0% agreement for nucleocapsid protein and 92.3% for spike protein. Therefore, individuals showing the positive response for antibodies measured in these assays also have neutralising antibodies. This grants a deeper understanding of the immune response to SARS-CoV-2 with the presence of binding antibodies that alert the immune system to the virus and neutralisation antibodies that prevent SARS-CoV-2 from binding to cells and protect them from attack. Both the N and S assay tests have high sensitivity and specificity ^16, 17^, but they do require that sufficient blood samples be provided. Given that samples were collected by participants at home, the ‘void’ rates are higher than would be expected. However, amongst those tested, the false positive/negative rates will be low.

Fourth, most tests (97%) were completed within approximately a two-week period in late April 2021, providing a snap-shot of antibody response at this time (although as ~3% of samples were returned in May or June 2021 these may not be wholly comparable due to the continuing roll-out of vaccinations and changing COVID-19 developments; see Table 8 for further exploration of this). Furthermore, as detailed in the introduction, this data collection has been co-ordinated as part of the LH&W NCS programme in collaboration with ten other cohort studies ( Table 1), allowing for cross-cohort comparisons to explore differences between cohorts and boost statistical power.

**Table 8.** coronavirus disease 2019 (COVID-19) antibody test results, stratified by date sample returned. Differences assessed using chi-squared test; n (%). A positive N (nucleocapsid) result is defined as an N response value of COI ≥ 1. A positive S (spike) result is defined as an S response value of U/ml ≥ 0.80. Participants with ‘void’ test results have been excluded here. Note that G1 participants were more likely to return a sample in May or June 2021, compared to G0 participants (G1: 81/1,850 [4.4%]; G0: 75/1,772 [2.7%]; *χ* ^2^(1, *n* = 4,622) = 9.52, *p* = 0.002); as antibody profiles differ between the generations, this may bias the associations below for the whole sample. We therefore also present the antibody test results stratified by both date sample returned and generation. Although sample sizes are small, these results suggest little association between date completed and either test result among G1 participants; for G0 participants, no association between date and S antibody response was found, although G0 participants who returned a sample in May/June were somewhat more likely to have a positive N antibody result. However, we urge caution in interpreting these results, as it is possible that these results are biased either by additional unmeasured confounding (e.g., by socioeconomic position, occupation, ethnicity) or by the small sample sizes introducing random error.

	Positive result	Negative result	*p*-value
**Whole sample** N antibody response ( *n* = 4,199) April 2021 May/June 2021 S antibody response ( *n* = 4,241) April 2021 May/June 2021	475 (11.7%) 18 (14.3%) 3,087 (75.1%) 85 (66.4%)	3,598 (88.3%) 108 (85.7%) 1,026 (24.9%) 43 (33.6%)	0.368 0.026
**Generation 0** N antibody response ( *n* = 2,526) April 2021 May/June 2021 S antibody response ( *n* = 2,555) April 2021 May/June 2021	238 (9.6%) 18 (14.3%) NA (>96.0%) ^1^ NA (>92.0%)	2,228 (90.4%) 108 (85.7%) NA (<4.0%) NA (<8.0%)	0.071 0.395
**Generation 1** N antibody response ( *n* = 1,673) April 2021 May/June 2021 S antibody response ( *n* = 1,686) April 2021 May/June 2021	237 (14.7%) 8 (12.1%) 685 (42.3%) 24 (36.4%)	1,370 (85.3%) 58 (88.9%) 935 (57.7%) 42 (63.6%)	0.554 0.340

^1^NA: Not Applicable - Actual numbers withheld due to small cell counts and potential disclosure risk (cell counts < 5).

These data also have limitations and issues of interpretation, many of which have been detailed above, which users of this resource should consider when using this data, such as: waning antibody responses potentially resulting in some false negative results ^18, 20^, especially as the length of time since infection in which the assay remains sensitive to pick up an antibody response is unknown, with >250 days post PCR confirmation potentially leading to a lack of antibodies being detected; problems when comparing results from different tests (i.e., differences in sensitivities between LFT and ECLIA tests); and issues of temporality, such as different data being available at different times which may not be comparable (e.g., assuming that a negative result in the past means that person has not caught the virus since). However, despite these challenges, as noted above we are able to triangulate this data against other sources in an attempt to somewhat verify these results ^13^, as will be described in more detail in forthcoming publications from the LH&W NCS.

Additionally, although not explored directly in this data note, previous ALSPAC data notes ^8– 11^ and other research ^24^ has established that invitation and response to ALSPAC’s COVID-19 data collection is non-random (e.g., with participants who are older, female, white and from higher socioeconomic backgrounds – among other factors – more likely to be invited and respond). Analyses using this data may therefore potentially be subject to selection bias, which may bias both prevalence estimates in this population and associations between variables ^25– 27^. These risks of selection bias are also present for the PHE Pillar I and II testing data, as who gets tested is known to be biased ^28, 29^. Additionally, as noted above, the proportion of positive spike protein antibodies reported here (~75%) is higher than that reported by the UK ONS on approximately the same dates in England as a whole (~65–70% from the UK Health Security Agency sero-surveillance programme; ~55–60% from the Coronavirus Infection Survey). Whether this is due to differences in demographics (e.g., the ALSPAC sample being older than the population as a whole), ALSPAC participants being more likely to have had COVID-19 and/or the vaccine, or other reasons, requires further investigation. Researchers should be aware of these potential biases, and explore them accordingly, when using this data. Despite these complexities, longitudinal population studies are vital in understanding the impact of the pandemic and the associated pre-pandemic risk factors.

In summary, this second antibody test data collected approximately one year since the start of the COVID-19 pandemic provides a picture of the immune response to COVID-19 infection and vaccination, and will enable researchers to explore patterns of both antibody response in this cohort to further understand patterns of infection and vaccination. These data are available for researchers as described below.

## Data availability

### Underlying data

ALSPAC data access is through a system of managed open access. The steps below highlight how to apply for access to the data included in this data note and all other ALSPAC data. The datasets presented in this article are linked to ALSPAC project number B3715, please quote this project number during your application. The ALSPAC variable codes highlighted in the dataset descriptions can be used to specify required variables.

1. Please read the ALSPAC access policy ( www.bristol.ac.uk/media-library/sites/alspac/documents/researchers/data-access/ALSPAC_Access_Policy.pdf) which describes the process of accessing the data and samples in detail, and outlines the costs associated with doing so.

2. You may also find it useful to browse our fully searchable research proposals database ( https://proposals.epi.bristol.ac.uk/?q=proposalSummaries), which lists all research projects that have been approved since April 2011.

3. Please submit your research proposal ( https://proposals.epi.bristol.ac.uk/) for consideration by the ALSPAC Executive Committee. You will receive a response within 10 working days to advise you whether your proposal has been approved.

If you have any questions about accessing data, please email alspac-data@bristol.ac.uk.

Please note that a standard COVID-19 dataset will be made available at no charge (see description below); however, costs for required paperwork and any bespoke datasets required additional variables will apply.

*COVID-19 Second Antibody Test Data File*

Data from the second ALSPAC COVID-19 antibody test is available in two ways.

1. A freely available standard set of data containing *all* participants together with key sociodemographic variables (where available) is available on request (see data availability section). This dataset also includes data obtained from the previous COVID questionnaires and first home-based antibody test. Subject to the relevant paperwork being completed (costs may apply to cover administration) this dataset will be made freely available to any bona fide researcher requesting it. Variable names will follow the format *sero_xxxx* where *xxxx* is a four-digit number. A full list of variables released is available here: https://doi.org/10.17605/OSF.IO/5QPCK. Frequencies of variable and details of any coding/editing decisions and derived variables are also available in the data dictionary: https://doi.org/10.17605/OSF.IO/5QPCK.
2. Formal release files have been created for G0 mothers, G0 fathers and G1 participants in the usual way and now form part of the ALSPAC resource (due to the small number of G1 partners contributing we will not be formally releasing this data, however, it may be available on request for specific G2 projects). These datasets (or sections therein) can be requested in the usual way. Variable names will replicate those in 1) above but as each variable in ALSPAC is uniquely defined we have added markers to denote the source of the variable. For example, in the second serology test dataset, the age of the participant at completion (in years) is denoted by *sero_9650*. In the G0 mother’s dataset this will be denoted by *serom_9650*, for G0 fathers/partner this will be *serop_9650* and for the G1 generation it will be *seroyp_9650*. Frequencies for all variables for each participant group are available in the data dictionary in the usual way ( http://www.bristol.ac.uk/alspac/external/documents/ALSPAC_DataDictionary.zip).

### Extended data

Open Science Framework: Second Serology testing. https://doi.org/10.17605/OSF.IO/5QPCK ^15^.

This project contains the following extended data:

1. VariableList_SERO.pdf (List of variable names and labels)
2. SERO_ALL_1a.pdf (Associated data dictionary including frequencies of all variables that are available)
3. Protocol_ALSPAC_SERO_v1.1.pdf (Study protocol)
4. ALSPAC PIS v1.1 Antibody_substudy.pdf (Participation information sheet given to participants about the antibody research study)
5. Instructions leaflet.pdf (Instruction leaflet given to participants detailing how to take and return the blood sample)

Data are available under the terms of the Creative Commons Attribution 4.0 International license (CC-BY 4.0).

## Ethical approval and consent

Participants consented electronically to take part in the antibody testing. Ethical approval for the study was obtained from the ALSPAC Ethics and Law Committee and the Local Research Ethics Committees. The East of England – Cambridge South Research Ethics Committee provided specific approval for this data collection (REC reference number: 21/EE/0061). Informed consent for the use of data collected via questionnaires and clinics was obtained from participants following the recommendations of the ALSPAC Ethics and Law Committee at the time. Study participants have the right to withdraw their consent for elements of the study or from the study entirely at any time. Full details of the ALSPAC consent procedures are available on the study website.
