# Supplementary material for: The Avon Longitudinal Study of Parents and Children - A resource for COVID-19 research: Antibody testing results, April – June 2021
Source: Wellcome Open Res. 2022 Jan 24;6:283. Originally published 2021 Oct 20. [Version 2] doi: 10.12688/wellcomeopenres.17294.2 (PMC8738971; doi:10.12688/wellcomeopenres.17294.2)
Supplement: Peer review history

# Peer review: Reviewer response for version 2

**DOI:** 10.21956/wellcomeopenres.19485.r48184

**Published:** 2022-01-24

**Authors:** Alicia Matijasevich

**Affiliations:** 1 Departamento de Medicina Preventiva, Faculdade de Medicina FMUSP, Universidade de São Paulo, São Paulo, Brazil

No further comments. I am glad with the responses.

Are sufficient details of methods and materials provided to allow replication by others?

Yes

Is the rationale for creating the dataset(s) clearly described?

Yes

Are the datasets clearly presented in a useable and accessible format?

Yes

Are the protocols appropriate and is the work technically sound?

Yes

Reviewer Expertise:

Epidemiology. Statistics.

I confirm that I have read this submission and believe that I have an appropriate level of expertise to confirm that it is of an acceptable scientific standard.

# Peer review: Reviewer response for version 1

**DOI:** 10.21956/wellcomeopenres.19121.r47542

**Published:** 2022-01-06

**Authors:** Matthias Pierce

**Affiliations:** 1 School of Health Sciences, Faculty of Biology, Medicine and Health, University of Manchester, Manchester, UK

The paper details antibody testing for two generations of the prospective cohort study ALSPAC. It provides some initial statistics and some comment on the quality of the data. Overall it is very clear and well written, and I have only a few minor suggestions.

**Abstract: **

- During my first few readings, it looked like 4,241 were void, I suggest rephrasing.

**Methods:**

- Figure 1 should show the overall N of the ALSPAC sample.

**Key results:**

- What are the comparisons between the sample demographic and the population demographics? In terms of education, or other SES markers.
- The proportion of positive results seems high, how does this compare to other published statistics? E.g. ONS

**Discussion:**

- One of the drawbacks for this study is the non-response – how do you plan on addressing this in analysis? E.g. through weighting/multiple imputation?

Are sufficient details of methods and materials provided to allow replication by others?

Yes

Is the rationale for creating the dataset(s) clearly described?

Yes

Are the datasets clearly presented in a useable and accessible format?

Yes

Are the protocols appropriate and is the work technically sound?

Yes

Reviewer Expertise:

Biostatistics, epidemiology with expertise in mental health.

I confirm that I have read this submission and believe that I have an appropriate level of expertise to confirm that it is of an acceptable scientific standard.

# Response

**Published:** 2022-01-14

**Authors:** Daniel Smith

**Affiliations:** University of Bristol, UK

We thank the reviewer for their positive and constructive comments, and we hope that our revisions have addressed any remaining queries/suggestions. The reviewer’s comments are below, followed by our responses in italics.

 Abstract:

- During my first few readings, it looked like 4,241 were void, I suggest rephrasing.

*We have changed this from “Of participants with a valid spike protein antibody test result (4,241; 8.2% void)…” to “Of 4,241 participants with a valid spike protein antibody test result (8.2% were void)…”. We have edited the nucleocapsid results accordingly, as well.*

 Methods:

- Figure 1 should show the overall N of the ALSPAC sample.

*As the overall N of ALSPAC has changed substantially over the last 30 years (we politely refer the reviewer to our key cohort profile papers: Boyd et al., 2013; Fraser et al., 2013; Northstone et al., 2019; Lawlor et al., 2019 [references 4 to 7 in the data note]), we feel it is more appropriate to add one layer showing the number of participants who were available to be approached to take part in our COVID research, i.e. those with a valid email address, who were still alive and had not withdrawn consent to be contacted. We have updated figure 1 accordingly.*

 Key results:

- What are the comparisons between the sample demographic and the population demographics? In terms of education, or other SES markers.

*As this question has been explored in depth in previous ALSPAC COVID-19 data notes and other research, we did not want to repeat this information in the present publication. However, we agree it is important point, and in our original submission noted in the discussion that “although not explored directly in this data note, previous ALSPAC data notes ^8–11^ and other research ^24^ have established that invitation and response to ALSPAC’s COVID-19 data collection is non-random (e.g., with participants who are older, female, white and from higher socioeconomic backgrounds – among other factors – more likely to be invited and respond).” We hope this is satisfactory.*

- The proportion of positive results seems high, how does this compare to other published statistics? E.g. ONS

*This is a great idea, and we thank the reviewer for suggesting it. We have now compared these ALSPAC results against statistics for England published by the Office for National Statistics (ONS), and added sections in both the methods and the discussion:*

*Methods: “Compared to nationwide antibody statistics compiled by the UK’s Office for National Statistics (ONS) on approximately the same dates (mid/late April 2021) in England, the proportion of positive spike protein antibodies reported here (~75%) is similar, albeit slightly higher, than that reported by the UK Health Security Agency sero-surveillance programme using the same Roche S antibody test (~65-70%). These ALSPAC results are also higher than the Coronavirus Infection Survey statistics, which used an ELISA test for immunoglobulins IgG based on SARS-CoV-2 trimeric spike protein, where antibody rates were ~55-60%.”*

*Discussion: “Additionally, as noted above, the proportion of positive spike protein antibodies reported here (~75%) is higher than that reported by the UK ONS on approximately the same dates in England as a whole (~65-70% from the UK Health Security Agency sero-surveillance programme; ~55-60% from the Coronavirus Infection Survey). Whether this is due to differences in demographics (e.g., the ALSPAC sample being older than the population as a whole), ALSPAC participants being more likely to have had COVID-19 and/or the vaccine, or other reasons, requires further investigation.”*

 Discussion:

- One of the drawbacks for this study is the non-response – how do you plan on addressing this in analysis? E.g. through weighting/multiple imputation?

*This depends on the research question being asked and the causal assumptions made, but yes, methods such as inverse-probability weighting, multiple imputation and/or sensitivity analyses could be applied to address potential selection bias due to non-response. However, we do not want to dictate how researchers analyse this data, as there is no ‘one-size-fits-all’ approach and the methods used to address this potential bias will depend on the specific research question.*

# Peer review: Reviewer response for version 1

**DOI:** 10.21956/wellcomeopenres.19121.r46578

**Published:** 2021-11-29

**Authors:** Alicia Matijasevich

**Affiliations:** 1 Departamento de Medicina Preventiva, Faculdade de Medicina FMUSP, Universidade de São Paulo, São Paulo, Brazil

This is an excellent manuscript that presents the results of antibody tests performed on ALSPAC cohort participants and describes results according several participants' characteristics. The manuscript is very well written, analyzed and discussed. Congratulations to the authors and the ALSPAC team for once again demonstrating pioneering research. Congratulations also for the high response rate that ALSPAC obtained.

 I would have liked the opportunity to read some information in the manuscript that could be useful to other researchers interested in the topic:

1. Blood collection procedure (instructions that the researchers sent to the participants). Also more information is needed about participants' expenses for the extraction of blood and sending the samples.
2. Is there any way to test a small sample to make sure the exams match the participants? This information could be included in the supplementary material.

Are sufficient details of methods and materials provided to allow replication by others?

Yes

Is the rationale for creating the dataset(s) clearly described?

Yes

Are the datasets clearly presented in a useable and accessible format?

Yes

Are the protocols appropriate and is the work technically sound?

Yes

Reviewer Expertise:

Epidemiology. Statistics.

I confirm that I have read this submission and believe that I have an appropriate level of expertise to confirm that it is of an acceptable scientific standard.

# Response

**Published:** 2022-01-14

**Authors:** Daniel Smith

**Affiliations:** University of Bristol, UK

We are delighted with the incredibly positive review and thank the reviewer for their constructive comments.

 Regarding the first part of point 1, the blood collection procedure instructions given to participants is part of the study protocol presented in the extended data. We have clarified this on page 5.

 For the second part of point 1, participants did not incur any expense for either blood extraction or sending the samples. We have included an additional sentence on page 5 addressing this: *“Participants incurred no cost in providing a blood sample, with the blood sample collection kit including all necessary instructions and equipment, and a cardboard box with a pre-paid form to return the samples in”.*

 Regarding point 2, unfortunately we are not clear what the reviewer may be referring to here. We would be very happy to respond with clarification.
